# Extracorporeal Shock Wave Therapy versus laser therapy in treating musculoskeletal disorders: a systematic review and meta-analysis

**DOI:** 10.1007/s10103-025-04392-0

**Published:** 2025-04-15

**Authors:** Mariam Ismail Hassan, Marwa Shafiek Mustafa Saleh, Mariam Hesham Sallam, Hadel Hesham Elkhodary, Mazen Mohamed Sayed, Haidy Samy, Afnan Hesham Mohamed, Ahmed Said Ashour, Esraa Mohamed Mosaid, Manar Hassan Zaghloul, Esraa Ramadan Elbathesh, Hina Vaish, Alshehri Mohammed Abdullah A, Ahmed Ibrahim Abdelhamed

**Affiliations:** 1 College of Physical Therapy, Misr University for Science and Technology, Giza, Giza, Egypt; 2 Faculty of Physical Therapy, Cairo University, Giza, Giza, Egypt; 3 Department of Physical Therapy, Faculty of Applied Medical Sciences, Al‐Zaytoonah University of Jordan, Amman, Amman, Jordan; 4 Faculty of Physical Therapy, Modern University for Technology and Information, Cairo, Cairo, Egypt; 5 School of Health Sciences, Chhatrapati Shahu Ji Maharaj University, Kanpur, Kanpur, India; 6 College of Applied Science, Physical Therapy, Taif University, Taif, Saudi Arabia

**Keywords:** Soft tissue disorders, Shockwave therapy, Photobiomodulation, Rehabilitation, Pain management, Functional recovery

## Abstract

This systematic review aimed to compare the effectiveness of Extracorporeal Shock Wave Therapy (ESWT) versus Laser therapy (Low-Level Laser Therapy (LLLT) and High-Intensity Laser Therapy (HILT) in treating musculoskeletal disorders (MSKDs). Systematic searches of randomized clinical trials (RCTs) were performed in six databases from inception till February 2025. Two researchers independently screened titles, abstracts, and full-text articles. Eligible studies had to report at least one of the following outcomes: pain, functionality, strength, range of motion (ROM), and quality of life (Qol). Risk of bias was assessed using the revised Cochrane Collaboration tool (RoB 2.0). Meta-analysis was conducted using Review Manager software version 5.4.1, and quality of evidence was evaluated using the GRADE approach. Twenty-eight RCTs involving 1,460 patients were included. The findings indicated that neither LLLT nor HILT showed a significant difference from ESWT in pain, strength, ROM, or quality of life. However, ESWT demonstrated a marginal statistically significant functional advantage over LLLT, but not over HILT. The GRADE certainty rating was rated as very low to moderate. According to GRDAE certainty rating, ESWT and laser therapies (LLLT and/or HILT) had equivalent effect in improving pain, strength, ROM, and Qol in patients with MSKDs, while ESWT had some short-term effects in improving functions more than LLLT but not than HILT. Large RCTs with higher methodological quality are needed to draw more incisive conclusions.

**Supplementary Information:**

The online version contains supplementary material available at 10.1007/s10103-025-04392-0.

## Clinical trial number

Not applicable.

## Introduction

Musculoskeletal Disorders (MSKDs) are among the most disabling and costly health issues in the modern era. These conditions may stem from acute injuries, repetitive strain, or acquired pathological processes, and are characterized by chronic pain, functional impairment, reduced mobility, and low quality of life (Qol) [1]. In 2020, MSKDs became the second-leading cause of non-fatal disability, affecting over 1.63 billion people globally [2]. Given the negative consequential impacts and the substantial burden on the healthcare system and economy, rehabilitation treatments for MSKDs have always held the spotlight within academic communities.

Physical therapy rehabilitation treatment offers a wide range of electrotherapeutic modalities that are clinically available to manage patients with MSKDs, such as Transcutaneous Electrical Nerve Stimulation (TENS), interferential therapy (IFT) [3], and ultrasound.

(US) [1, 4]. Advantageously, Extracorporeal Shock Wave Therapy (ESWT) and laser Therapies are evidenced modalities to reduce pain and improve functional ability in various MSKDs [5–9].

ESWT is a non-invasive modality that utilizes high-energy acoustic waves, lasting for a microsecond on a target-affected body area [10]. It is theorized to regenerate tissues through increasing the protein synthesis by the mechanical load exerted on the affected tissue. It is also hypothesized to relieve pain through two mechanisms: one suggests that ESWT may stimulate endorphins release, causing analgesia, and modulating pain signals—a process known as hyperstimulation. The other proposes that ESWT may cause damage to the nerve fibers, reducing pro-inflammatory mediators and, thereby, pain level [11].

On the other hand, laser therapies, including Low-Intensity Laser Therapy (LLLT) and High-Intensity Laser Therapy (HILT), are two physical agents widely used with different MSKDs, each with a distinct mechanism. LLLT is a class III laser, operating at power output ≤ 500 mW. Relying primarily on photobiomodulation, LLLT stimulates cellular processes, promotes tissue regeneration, alleviates pain (through neural blockade), and reduces inflammation [12], while HILT is a Class IV laser, operating at power levels > 500 mW. Combining photobiomodulation and a thermal effect, HILT facilitates the ability to penetrate deeper into the tissues, producing analgesic and anti-inflammatory effects [13].

Recent studies regarding ESWT and laser therapies (HILT and/or LLLT) indicate that both modalities are safe and effective in treating various MSKDs. However, previous systematic reviews comparing both treatments have not reached a common ground, and there is still inconsistency between their results in determining which method is best for managing different MSKDs. Previous systematic reviews conducted by Karanasios et al., 2021 and Charles et al., 2023 comparing the effectiveness of ESWT to laser suggest that ESWT might not be superior to LLLT in reducing pain in the short term nor improving functionality in cases of chronic lateral epicondylitis (LE) [14] and plantar fasciitis (PF) [15]. However, the number of trials adopted in the analyses was relatively small (only two and five trials, respectively). Moreover, a recent systematic review reported no difference between ESWT and HILT in reducing pain intensity at three months follow-up in cases of PF [16]. Conversely, a more recent systematic review by Ferlito et al., 2023 suggests that LLLT is superior to ESWT in reducing pain intensity in the short-term follow-up in patients with PF, demonstrating a large effect size (MD = − 20.94) [17].

Based on the foregoing, there is still inconsistency between systematic reviews comparing ESWT and LLLT, despite both modalities seem able to produce desirable outcomes in various MSKDs. In addition, limited evidence exists comparing ESWT to HILT, unlike LLLT. Recent trials have also emerged since those referenced in previous reviews. However, no systematic review has been conducted to date directly comparing ESWT to both types of laser therapy in the management of all MSKDs.

Comparing ESWT and laser therapies might be critical to optimizing patient outcomes and guiding clinical decision-making, as they are widely used in rehabilitation. Hence, this review aimed to systematically investigate the current evidence of ESWT directly compared to laser therapy (i.e., HILT and/or LLLT) primarily on reducing pain and improving function, and secondarily in enhancing strength, quality of life, and ROM for patients with MSKDs at short-(≤ 12 weeks), mid-(> 12 weeks and ≤ 6 months), and long-(> 6 months) term follow-ups. We hypothesize that there would be a difference between ESWT and laser therapies in the outcomes assessed.

## Methods

### Protocol and registration

The protocol of this systematic review was preregistered in the International Prospective Register of Systematic Reviews (PROSPERO) database (CRD42024566599), and it was reported according to the Preferred Reporting Items for Systematic Reviews and Meta-Analyses guidelines (PRISMA 2020) [18].

### Eligibility criteria

We assessed the eligibility criteria based on the participants, interventions, comparators, outcomes, and study design (PICOS) approach [19]: (1) Population: both genders aged ≥ 18 years diagnosed with MSKDs experiencing pain and/or ROM restriction or functional disability. (2) Interventions: ESWT alone or combined with Traditional treatment. (3) Comparison: Laser therapy alone or combined with Traditional treatment. (4) Outcomes: pain intensity, functional status, ROM, strength, and quality of life. Study design: RCTs. We excluded non-English-language studies, cross-sectional studies, cohort studies, case reports, review articles, editorials, conference abstracts, protocol registrations, and non-human studies.

### Literature searching

We conducted a comprehensive database search using PubMed, Cochrane Central Registration of Clinical Trials (CENTRAL), Web of Science (WOS), Scopus, Google Scholar, and PEDro databases from inception till June 2024 and updated in February 2025. We checked the reference lists of included articles to identify additional studies. The search strategy included MeSH and entry terms related to ESWT and laser therapy such as: “Extracorporeal shockwave therapy”, “ESWT”, “shock wave therapy”, “Laser Therapy”, “Photobiomodulation”, “low level laser therapy”, “high intensity laser therapy”. Population terms were not included to enhance the sensitivity of the search. The detailed search strategies are available in Online Resource 1.

### Study selection

After the initial database search, we imported the identified articles into Mendeley software and checked for duplicates. After removing duplicates, two authors separately screened the titles and abstracts of the remaining articles. Articles that met the inclusion criteria were retrieved for full-text reading by two different authors. The authors then compared the studies, and if there were any disagreements, they discussed and reached a consensual decision.

### Data extraction sheet

Two authors independently extracted data from eligible studies into a pre-designed data collection form using Microsoft Excel. The following variables were summarized in the sheet: author’s name, year of publication, participant characteristics (number, gender, and mean age), ESWT characteristics (frequency, impulses, energy flux density, intensity, total number of sessions, number of sessions per week, type, and application site), laser therapy characteristics (wavelength, power, energy density per session, total number of sessions, number of sessions per week, type, source, pulse mode, and application site), co-intervention, control interventions, time of treatment, points of assessment, outcome measures, loss to follow-up and adverse effects. Discrepancies between reviewers were solved through discussion, and the senior author was consulted when consensus could not be reached.

### Risk of bias assessment

Two independent authors assessed the risk of bias using the Cochrane Risk of Bias tool for randomized trials (RoB 2.0) [20]. This tool evaluates five domains: bias arising from the randomization process, bias due to deviations from the intended intervention, bias due to missing outcome data, bias in the measurement of the outcome, and bias in the selection of the reported result. The two authors independently judged each item as either ‘low risk’, high risk’, or ‘some concerns’. In cases of disagreement, the issue was resolved through discussion. If disagreement persisted, the senior author was consulted to arbitrate.

### Statistical analysis

Statistical analysis was carried out using Review Manager (RevMan) software version 5.4 (The Nordic Cochrane Centre, Copenhagen, Denmark). Meta-analyses with forest plots were performed when two or more studies were available. The inverse variance method was used for analyzing all variables (pain, functionality, strength, and quality of life). We combined mean and standard deviation, and if data were represented as median and range, we converted them to mean and standard deviation using Wan’s method [21]. The effect size was reported as the Mean Difference (MD) or Standardized Mean Difference (SMD) if there was a difference in employed scales, both along with the 95% Confidence Interval (CI). We used a random effects model since the included studies were drawn from the literature. Heterogeneity was examined using Cochran’s Q statistic and I² (with statistical significance set at *p* < 0.10) used to describe the percentage of variability with I² values of < 50%, 50–75%, and > 75% representing low, medium, and high heterogeneity, respectively [22]. The effects were quantified at short- (≤ 12 weeks), mid-(> 12 weeks and ≤ 6 months), and long-(> 6 months) follow-ups.

### Quality assessment

The quality of evidence was assessed according to the Grading of Recommendations, Assessment, Development, and Evaluations (GRADE) system using the GRADEpro guideline development tool (GDT) platform (https://doi.org/gradeproorg. org). We assessed all outcomes across different follow-up periods, MSKDs, and intervention comparison [23]. The level of evidence was classified as high, moderate, low, and very low based on five items: risk of bias, imprecision, inconsistency, indirectness, and publication bias. Two reviewers independently performed the assessment, and a senior author was consulted if necessary.

## Results

### Study selection

A total of 4,807 records were identified (4,797 records from databases and ten records from reference and citation searching). Following the removal of duplicates, the remaining 3,356 records were selected for screening phase, where 3,296 records were excluded based on title/abstract screening, and 27 reports were excluded based on full-text screening, as shown in Online Resource 2. Finally, a total of 28 RCTs were included: 18 studies examined the efficacy of ESWT vs. LLLT [24–41], while ten studies examined the efficacy of ESWT vs. HILT [42–51].

From the included RCTs, ten studies about PF [24, 27, 29, 31, 34, 36, 42, 45–47], six about LE [25, 30, 35, 48–50], three about myofascial pain syndrome (MPS) [26, 28, 41], two about Carpal tunnel syndrome (CTS) [37, 38], three related to knee osteoarthritis (KOA) [32, 33, 44], two about shoulder impingement syndrome (SIS) [39, 43], one study focused on patients with calcaneal spur (CS) [40], and another one on patients with de Quervain tenosynovitis (DQT) [51]. Population characteristics of the included studies are available in Online Resource 3.

### Risk of bias

The risk of bias assessment across the 28 included studies revealed several areas of concern. Regarding the generation of randomization, only 17 studies [25, 27, 29, 32–34, 37, 38, 41–48, 51] demonstrated a low risk of bias, ten studies [24, 26, 30, 31, 35, 36, 39, 40, 49, 50] were classified as having some concerns, while one study [28] demonstrated a high risk. In terms of bias due to deviations from the intended interventions, six studies [25, 34, 44, 46, 50, 51] were assessed as low risk, while two study showed high risk [48, 50], and the rest showed some level of concern [24, 26–32, 35–43, 45, 47, 49]. Missing outcome data was less problematic, with 19 studies [24, 29, 30, 32–35, 37–39, 41, 43–49, 51] presenting a low risk, eight studies [25–27, 31, 36, 40, 42, 50] rated as some concerns, and only one study [28] exhibiting a high risk. The risk of bias related to the measurement of outcomes was categorized as having some concerns in all studies [24–33, 35–41, 43–45, 47–51], except for three studies that were rated as low risk [34, 42, 46]. Selection of the reporting bias was a significant issue, with 17 studies [25–28, 30–32, 34–36, 39, 40, 43, 44, 48, 49, 51] raising some concerns, six studies [33, 37, 41, 45, 47, 50] rated as low risk, and five studies [24, 29, 38, 42, 46] demonstrating a high risk. Overall, a thorough examination of bias dominance risks indicated that 16 articles [25, 27, 30, 32–35, 37, 39, 41, 43–45, 47, 48, 51] raised some concerns, whereas 12 articles [24, 26, 28, 29, 31, 36, 38, 40, 42, 46, 49, 50] exhibited a high level of risk. The detailed risk of bias assessment of all included RCTs is shown in online Resource 4.

### Participants

Overall, the 28 eligible studies were published between 2014 and 2025, encompassing a total of 1,460 participants, of whom 375 (26%) were men. It is noteworthy to emphasize that one study [43] did not provide gender information for its participants. The ages of the participants varied significantly, ranging from 22 to 73 years. Additionally, the review indicated that 93 (6.3%) participants were lost to follow-up, 58 in the ESWT vs. LLLT studies and 35 in the ESWT vs. HILT studies.

### Intervention

The effectiveness of ESWT versus laser therapy was compared either alone [27, 31, 39, 41, 46, 50, 51] or in conjunction with co-interventions, such as exercise programs [24–26, 28–30, 32–34, 36, 42, 47], traditional treatments [37, 38, 40, 43–45, 48, 49], or lateral epicondyle bandages [35]. All studies included two arms, except six studies with three arms [31, 34, 37, 43, 45, 49], and two study with four arms [29, 33].

Significant variations existed in the ESWT protocols across the included studies. Regarding ESWT type, radial was the most commonly reported, featured in 13 studies [24, 28, 32–35, 37, 38, 42, 43, 46–48]; whereas, focused ESWT was reported in only three studies [25, 29, 44], and 12 studies did not report the ESWT type used [26, 27, 30, 31, 36, 39–41, 45, 49–51]. The frequencies utilized ranged from three HZ to 21 HZ in 21 studies [26–29, 31–33, 35–38, 40, 41, 43, 45–51]; one study applied varying frequencies (12 MHZ and 15 MHZ) [42], while six studies did not specify the frequency employed [24, 25, 30, 34, 39, 44]. The treatment intensity varied widely, ranging from one bar to ten bar in 21 studies [24, 26, 27, 31, 33–43, 45–47, 49–51], while the resting studies did not provide details on the intensity applied [25, 28–30, 32, 44, 48]. Energy flux density values ranged from 0.02 mJ/mm² to 0.38 mJ/mm² in ten studies [24, 25, 29, 32, 34, 39, 41, 44, 47, 48]; one study applied varying energy densities of 3 J/m^2^ [28], while 17 studies did not report the density employed [26, 27, 30, 31, 33, 35–38, 40, 42, 43, 45, 46, 49–51]. Only one study did not specify the number of ESWT sessions per week [28], whereas the other studies varied from one to three sessions [24–27, 29–51].

Protocols for laser therapy (both LLLT and HILT) were heterogeneous regarding the reported parameters. For LLLT, wavelength ranged from 685 nm to 1064 nm in 16 studies [24–27, 29–40], while two studies did not provide details on the specific wavelengths used [28, 41]. For HILT, four studies employed a wavelength of 1064 nm [44, 46–48], one study used a wavelength of 980 nm [45], and five studies did not specify the wavelength used [42, 43, 49–51]. LLLT power settings varied widely between 30 mW and 100 mW in eight studies [24, 25, 27, 28, 31–34], while only one notable study reported a power output of 2000 mW [41], another one applied power 800 and 2000 mW [29], and the remaining eight studies did not disclose specific power levels [26, 30, 35–40]. HILT power settings ranged from 4 W to 30 W in eight studies [42, 45–51], while two studies did not specify the power utilized [43, 44]. For LLLT, energetic density varied between 1.2 J/cm^2^ and 8 J/cm^2^ in 13 studies [24–29, 31, 32, 34, 37, 38, 40, 41], while five studies applied varying energy across sessions [30, 33, 35, 36, 39]. For HILT, energy density values ranged from 5 J/cm² to 120 J/cm² study in six studies [42, 46, 48–51], while the other four studies utilized different energy levels [43–45, 47]. LLLT was administered across six to 15 sessions in 16 trials, and one study did not report the total number of sessions [40]. HILT trials applied a total of four to 15 sessions [42–51].

The follow-up periods varied across the studies, ranging from one to 16 weeks. The evaluation of adverse effects was explicitly documented in 11 out of the 28 studies; eight studies reported no adverse effects associated with the interventions being investigated [31, 35, 36, 39, 41, 45–47], while three studies documented the presence of adverse effects, including bruising [42], pain [25, 29], temporary reddening [29] after applying ESWT. Additional data on the intervention characteristics of the included studies are given in Online Resource 5.

### Outcome measures

A meta-analysis was conducted, including RCTs that consistently evaluated pain intensity, functionality, strength, and quality of life, while ROM was synthesized narratively due to a lack of comparability.

**Fig. 1:**
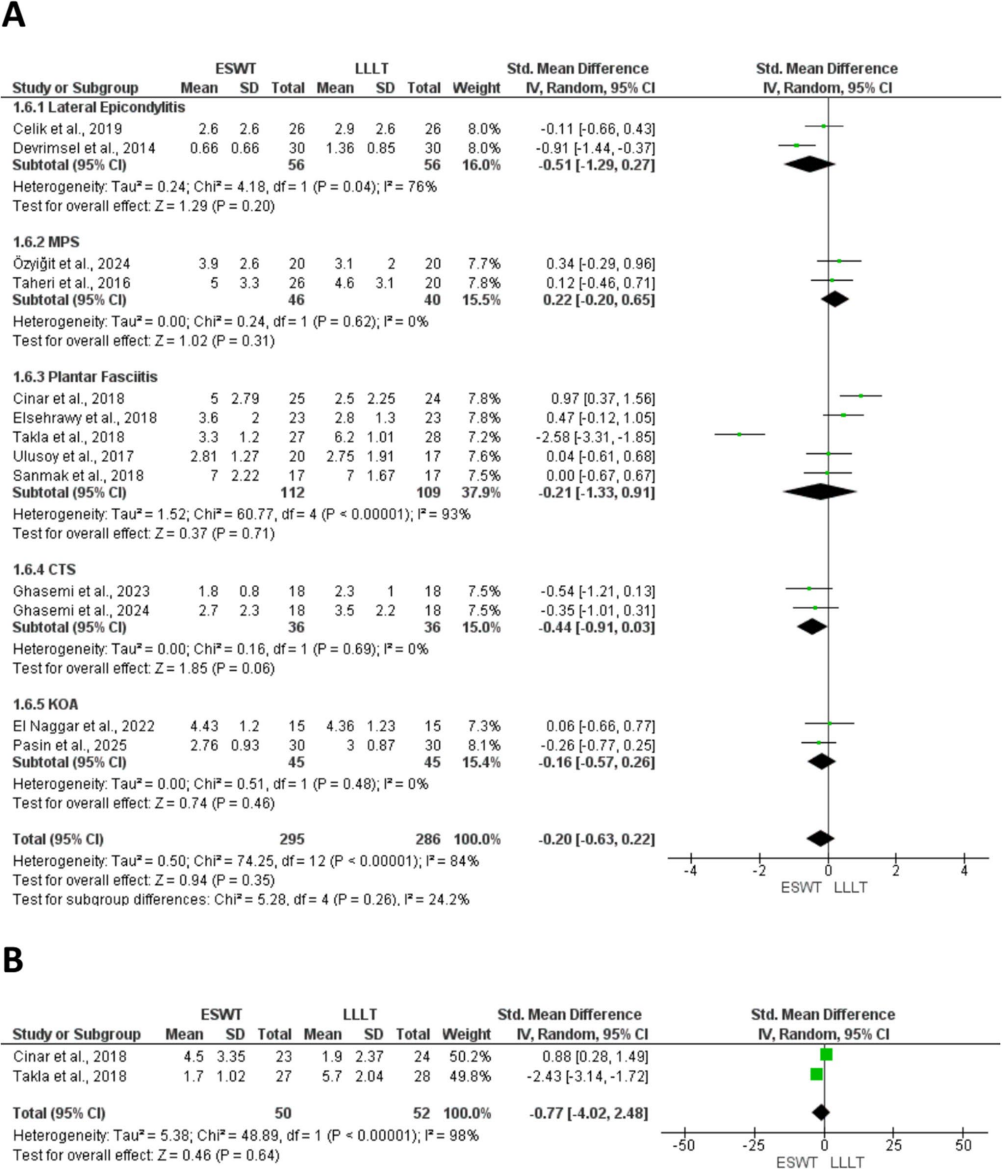
(1**A**) Forest plots of pain at short-term follow-up comparing ESWT vs. LLLT, (1**B**) and pain at medium-term follow-up comparing ESWT vs. LLLT

**Fig. 2:**
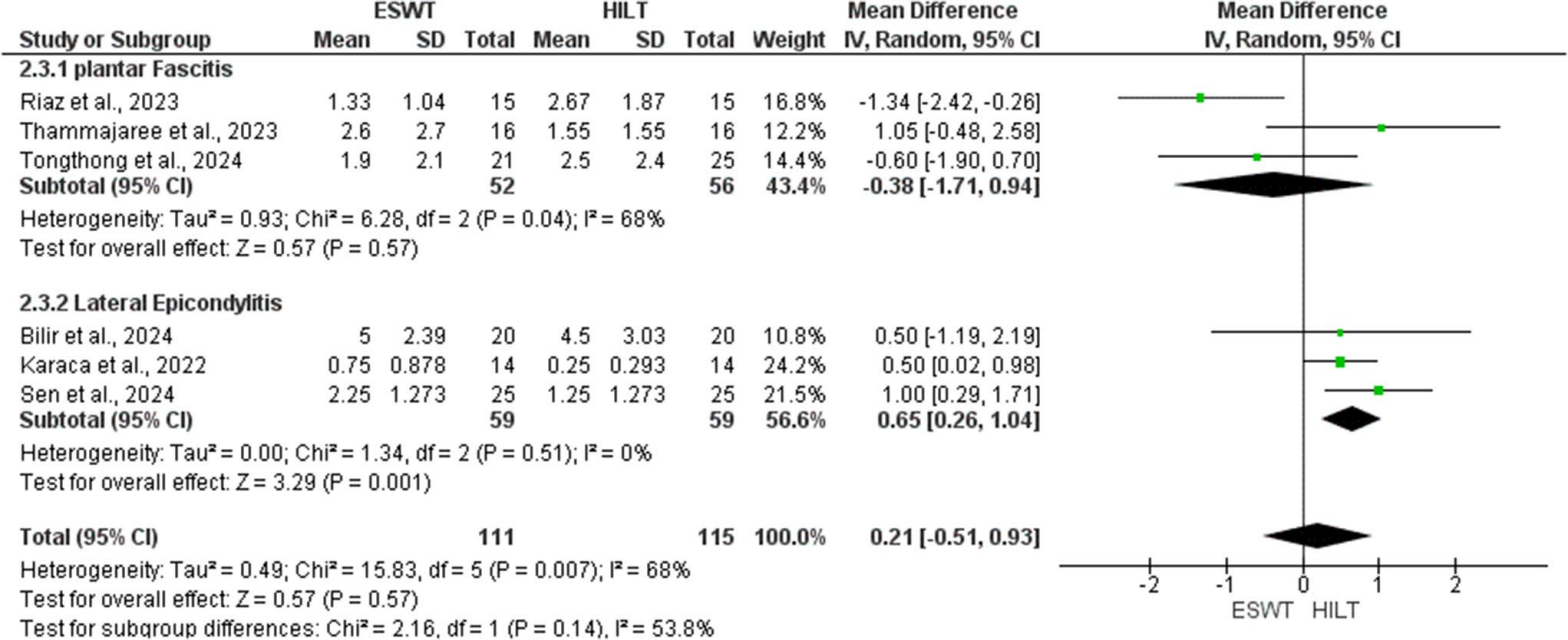
Forest plot of pain short- term follow-up comparing ESWT vs. HILT

**Fig. 3:**
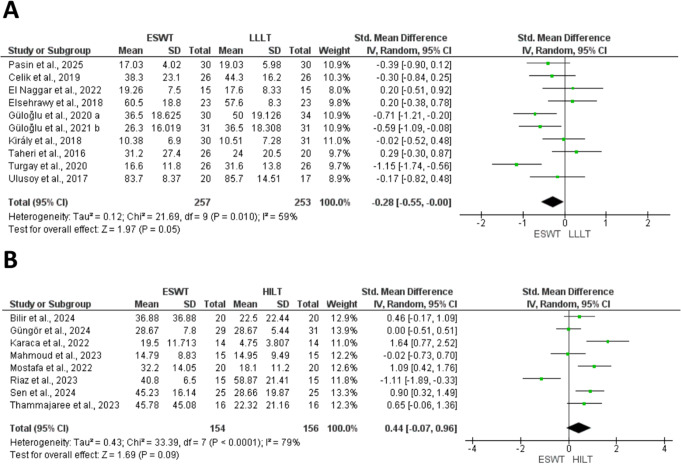
(**A**) Forest plots for the overall effect on short-term functionality comparing ESWT vs. LLLT, (**B**) and overall effect on short-term functionality comparing ESWT vs. HILT

**Fig. 4:**
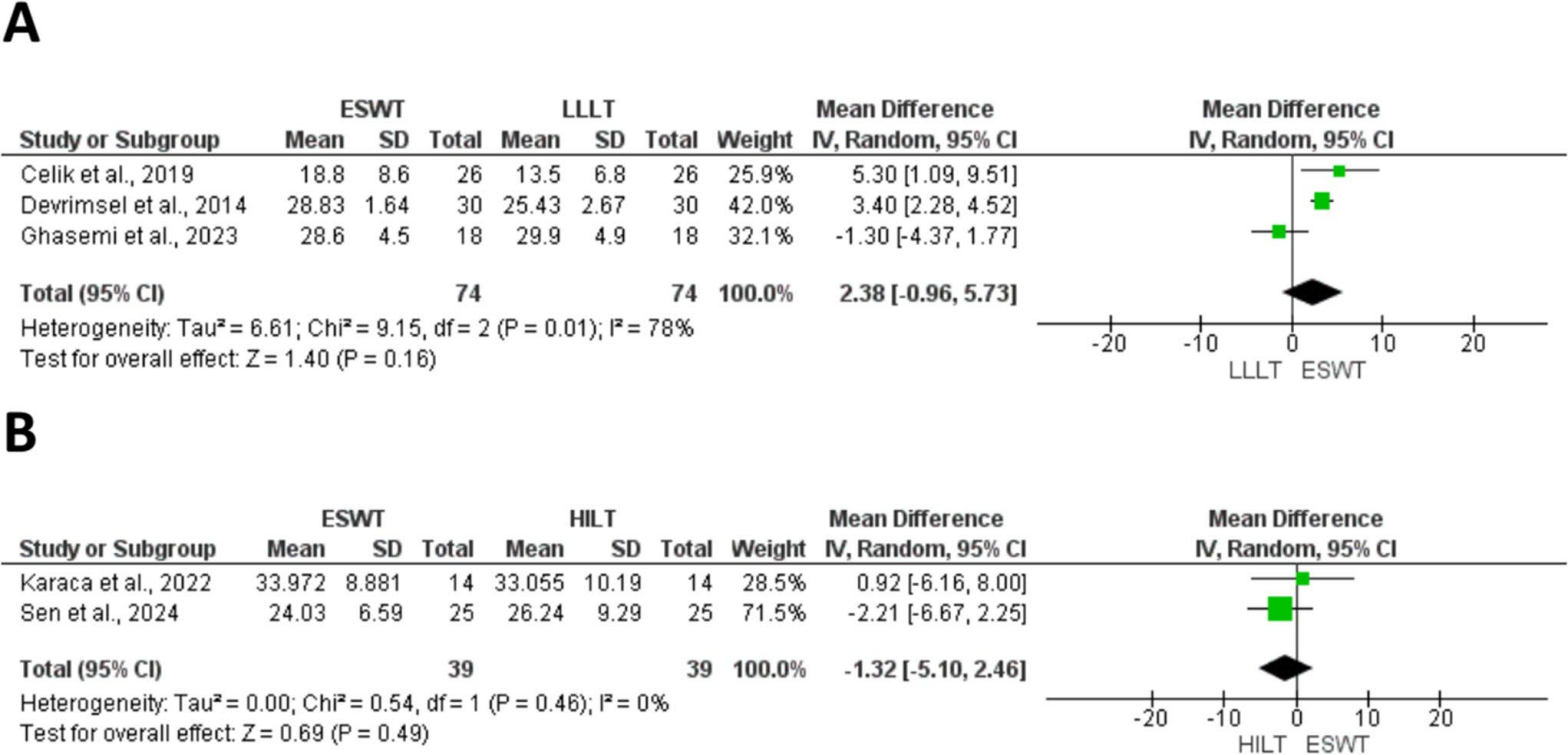
Forest plots of hand grip strength short-term follow-up comparing ESWT vs. LLLT (**A**), and hand grip strength short-term follow-up comparing ESWT vs. HILT (**B**)

**Fig. 5:**
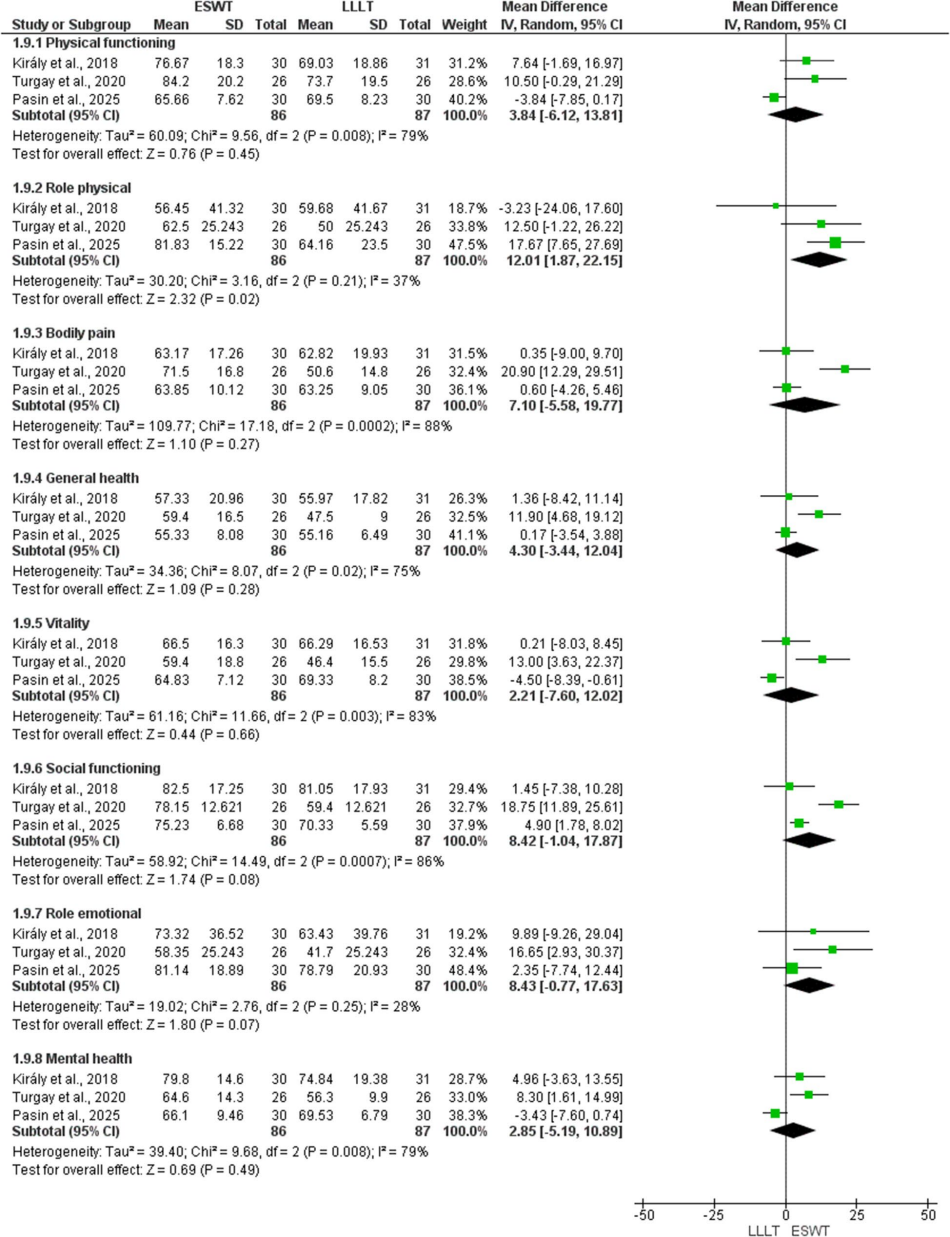
Forest plot of quality-of-life short-term follow-up comparing (ESWT) vs. (LLLT)

At this stage, two studies were excluded from quantitative synthesis. One study [42] was analyzed qualitatively, as it was the sole medium-term study comparing ESWT to HILT, with an emphasis on quality of life, whereas another study [24]` was entirely omitted from both qualitative and quantitative syntheses, as functional outcomes were presented only at the subscale level. As a result, 26 RCTs were eligible for meta-analysis. Figures 1A and B, 2, 3A and B and 4A and B, and 5 present meta-analyses result for the outcomes of interest. Results for both comparisons were statistically analyzed considering *p* ≤ 0.05.

## Pain

### Short-term follow-up

Thirteen studies assessed the pain score between ESWT and LLLT on short-term follow-up, using the Visual analogue scale (VAS) [25–29, 31–33, 35–38] and Numeric rating scale (NRS) [34] (Fig. 1A). There was no significant difference between ESWT and LLLT in terms of pain reduction in short-term follow up (SMD: -0.20; 95% CI: -0.63, 0.22, *p* = 0.35; I^2^ = 84%). In addition, the subgroup analysis revealed no significant difference between ESWT and LLLT in patients with LE (SMD: -0.51; 95% CI: -1.29, 0.27; *P* = 0.2; I^2^ = 76%), MPS (SMD: 0.22; 95% CI: -0.20, 0.65; *P* = 0.31; I^2^ = 0%), PF (SMD: -0.21; 95% CI: -1.33, 0.91; *P* = 0.71; I^2^ = 93%), CTS (SMD: -0.44; 95% CI: -0.91, 0.03; *P* = 0.06; I^2^ = 0%), and KOA (SMD: -0.16; 95% CI: -0.57, 0.26; *P* = 0.46; I^2^ = 0%).

Six studies assessed the pain score between ESWT and HILT on short-term follow-up, using VAS [45–50] (Fig. 2). There was no significant difference between ESWT and HILT in terms of pain reduction in short-term follow-up (MD: 0.21; 95% CI: -0.51, 0.93, *p* = 0.57; I^2^ = 68%). For the subgroup analysis, three studies [45–47] showed no significant difference between ESWT and HILT in patients with PF (MD: -0.38; 95% CI: -1.71, 0.94; *P* = 0.57; I^2^ = 68%), but three studies [48–50] revealed a statistically significant difference for the HILT in patients with LE (MD: 0.65; 95% CI: 0.26, 1.04; *P* = 0.001; I^2^ = 0%).

### Medium-term follow-up

Two studies [29, 34] assessed the pain score between ESWT and LLLT on medium-term follow-up, using VAS [29] and NRS [34] (Fig. 1B). Based on quantitative synthesis, there was no significant difference between ESWT and LLLT in patients with PF (SMD: -0.77; 95% CI: -4.02, 2.48; *P* = 0.64; I^2^ = 98%).

Only one study [42] compared the effect of ESWT on HILT in the medium-term follow-up. As per narrative synthesis, there was superiority of HILT over ESWT in reducing pain on VAS (*p* = 0.03).

.

### Functionality

#### Short-term follow-up

Ten studies [25, 28, 30–33, 36, 39–41] assessed the short-term effect of functionality between ESWT and LLLT, using Disabilities of the arm, shoulder, and hand (DASH) [25, 30], Western Ontario McMaster University Osteoarthritis Index (WOMAC) [32, 33], Foot function index (FFI) [36, 40], Neck disability index (NDI) [28, 41], Roles–Maudsley score (RMS) [31], and shoulder pain and disability index (SPADI) [39] (Fig. 3A). The analysis showed a marginal statistically significant difference for the ESWT (SMD: -0.28; 95% CI: − 0.55, -0.005; *P* = 0.05; I^2^ = 59%).

Eight studies [43–46, 48–51] investigated the short-term effect of ESWT compared to HILT on functionality, using FFI [45, 46], SPADI [43], 6-min walking test (DW6m) [44], Patient-Related Lateral Epicondylelitis Evaluation (PRTEE) [49], quick version (Q-DASH) [48, 50, 51] (Fig. 3B). The results show no significant difference between the two groups (SMD: 0.44; 95% CI: -0.07, 0.96; *P* = 0.09; I ^2^ = 79%).

### Secondary outcomes

#### Strength

##### Short-term follow-up

Three studies [25, 35, 37] assessed the short-term effect between ESWT and LLLT on grip strength through hand-held dynamometer (HHD) (Fig. 4A). The analysis showed no significant (MD = 2.38, 95% CI = -0.96 to 5.73, *P* = 0.16; I²= 78%).

Two studies were synthesized qualitatively due to heterogenous outcomes. They compared the short-term effect of ESWT to LLLT on the elbow flexors and extensors strength using HHD [25] and finger pinch strength using a finger dynamometer (*p* > 0.05) [37], reporting no significant differences between groups.

Two studies [48, 49] investigated the short-term effect of ESWT compared to HILT on hand grip strength using a dynamometer (Fig. 4B). The overall effect revealed no significant difference between the two groups (MD: -1.32; 95% CI: -5.10, 2.46; *P* = 0.49; I ^2^ = 0%)

### Quality of life

#### Short-term follow-up

Three studies [30, 33, 41] investigated the short-term effect of ESWT compared to LLLT on SF-36 domains (Fig. 5). ESWT was more effective than LLLT in improving only role physical (MD: 12.01; 95% CI: 1.87, 22.15; *P* = 0.02; I ^2^ = 37%). However, no significant difference was detected in the remaining items, namely physical functioning (MD: 3.84; 95% CI: -6.12, 13.81; *P* = 0.45; I ^2^ = 79%), bodily pain (MD: 7.10; 95% CI: -5.58, 19.77; *P* = 0.27; I ^2^ = 88%), general health (MD: 4.30; 95% CI: -3.44, 12.04; *P* = 0.28; I ^2^ = 75%), vitality (MD: 2.21; 95% CI: -7.60, 12.02; *P* = 0.66; I ^2^ = 83%), social functioning (MD: 8.42; 95% CI: -1.04, 17.87; *P* = 0.08; I ^2^ = 86%), role emotional (MD: 8.43; 95% CI: -0.77, 17.63; *P* = 0.07; I ^2^ = 28%), and mental health (MD: 2.85; 95% CI: -5.19, 10.89; *P* = 0.49; I ^2^ = 79%).

Only one study [42] compared the effect of ESWT to HILT on Qol in the medium term. Narratively analyzed due to a lack of comparability, the results revealed the superiority of HILT, in terms of the physical and mental components of SF-36 (*p* = 0.001, 0.008) respectively.

### Range of motion

#### Short-term follow-up

Range of motion measurements were reported in two studies using a goniometer [39] and electro goniometer [43]. Both studies were excluded from the meta-analysis due to high heterogeneity. Only one study investigated the effects of ESWT versus LLLT on shoulder ROM in patients with SIS [39], and found no significant difference between the two treatment groups in flexion (*p* = 0.077), abduction (*p* = 0.28), internal (*p* = 0.353) and external rotation ROM (*p* = 0.611). Also, only one study compared the effects of ESWT and HILT on shoulder flexion and abduction in patients with SIS [43], revealing no significant difference between groups.

### Quality of evidence

The GRADEpro GDT platform was used to assess the quality of evidence on the effect of ESWT versus laser therapies in treating MSKDs according to the GRADE criterion. Detailed GRADE evidence for all results is presented in **online Resource 6.**

### ESWT vs. LLLT

For pain, the evidence in the short-term follow-up was downgraded to low quality for MPS studies [26, 28], CTS studies [37, 38], and KOA studies [32, 33] and very low for LE studies [25, 35] and PF studies [27, 29, 31, 34, 36], while in the medium-term follow-up, the evidence of pain was downgraded to low in patients with PF. The evidence of functionality, quality of life, and grip strength in the short-term follow-up was downgraded to very low. The quality of evidence for short-term shoulder ROM was moderate.

### ESWT vs. HILT

The evidence for pain in the short-term follow-up was downgraded to low in patients with PF [45–47] and LE [48–50]. The evidence of functionality in the short-term follow-up was downgraded to very low. The evidence for grip strength and shoulder ROM in the short-term follow-up was downgraded to moderate. For the quality of life, the evidence in the medium-term follow-up was downgraded to moderate.

## Discussion

This systematic review and meta-analysis investigated the effect of ESWT versus laser therapy (LLLT and/or HILT) primarily on reducing pain and enhancing function, and secondarily on improving quality of life, strength, and ROM in patients with MSKDs. The findings suggest no statistically significant difference between ESWT and both types of laser therapies in improving pain, strength, ROM, or Qol in patients with MSKDs. However, ESWT showed a marginal statistical difference in improving functionality compared to LLLT but not to HILT. The GRADE rating of the results was very low to moderate, suggesting that the evidence was inadequate, and the results could thus not be confirmed.

The current meta-analysis found no significant difference between ESWT and LLLT or HILT in reducing pain in patients with MSKDs. Both ESWT and laser therapy are suggested to reduce pain from distinct mechanisms. LLLT is hypothesized to trigger secreting endogenous opioids (i.e., endorphins), blocking central pain, reducing mediators (i.e., histamine and bradykinin), and increasing pain threshold. It may also inhibit sensory nerve fibers, decreasing the transmission of pain signals. In addition, the HILT’s wavelength allows it to work with more focused and intense light energy, with a further increase in the concentration of endogenous chromophores during the treatment program [9]. Furthermore, HILT was found to induce the release of endorphins and serotonin at the peripheral nerve endings and decrease proinflammatory cytokines and other inflammatory mediators, such as interleukin-1, interleukin-6, prostaglandin, C-reactive protein, and tumor necrosis factor-alpha [52]. Moreover, HILT is reported to have a photothermal effect that increases the local tissue temperature and blood circulation in joints, promoting the exchange of nutrients in cartilage, stimulating tissue regeneration, and reducing pain, oedema, and inflammation [53]. A recent study emphasizes the benefits of laser therapy in reducing pain. Labanca et al., (2024) applied a multiwave locked system laser therapy on the whole lumbar region in patients with low back pain. The measured output energy was 5 J/cm2 over a 30 cm2 back area. Compared to sham laser therapy, laser therapy showed a significant pain reduction in pain measured on VAS at week seven (*P* = 0.045) posttreatment [54]. On the other hand, ESWT may increase collagen production, enhance cellular proliferation, and modulate pain pathways through gate control mechanisms, all of which may collectively contribute to reducing pain severity, and thereby, improving mobility, functionality, and Qol [11]. The findings of the current systematic review are consistent with previously published systematic reviews conducted by Charles et al. 2023 [15] and Karanasios et al. 2021 [14], to compare the short-term effect of ESWT and LLLT in treating PF and LE, respectively; they reported no significant difference between either intervention. Similarly, Li et al. 2018 compared the effect of ESWT and LLLT in patients with PF. Pairwise results showed that ESWT and LLLT significantly reduced pain versus placebo (0–6 weeks), but indirect comparison in network meta-analysis found no difference between them [55].

Regarding functional outcomes, meta-analysis shows the confidence interval barely crosses the threshold of statistical significance, indicating a marginal effect detected in favor of ESWT compared to LLLT in the short term. However, this observed difference may be influenced by heterogeneity, suggesting variability between studies that may stem from different populations and assessment methods. A recent systematic review conducted by Charles et al. 2023 [15] reported no statistically significant difference between ESWT and LLLT in improving functional outcomes in patients with PF in the short term based on low to moderate certainty evidence. Given our evidence was rated as very low, current results should be interpreted cautiously. There was also no short-term difference between ESWT and HILT in improving functionality. One possible explanation might be the lack of differences in pain improvement between the two treatment modalities, which was supported by a previous study conducted by Song et al. 2018 [56] who stated that pain control could be one of the main reasons for improving functional abilities in patients with MSKDs. The findings of the current study regarding the insignificant difference between ESWT and HILT in improving function contradict those of Arroyo-Fernandez et al., 2023 who found that HILT is more effective compared with control and other conservative treatments with potentially superior to ESWT in improving function in patients with MSKDs [57]. The contradiction between their systematic review and ours may be because they relied in their systematic review on only one study that compared HILT and ESWT out of all the included studies, while the present systematic review has included a larger study number, larger sample size, and covering a broad spectrum of MSKDs. However, the current results are still inconclusive due to the largely low quality of evidence.

Concerning quality of life, only one subscale of SF-36 questionnaire (Role Physical) has shown a significant difference in the short term between ESWT and LLLT, favoring ESWT, while the remaining seven subscales of Qol (Physical Functioning, Vitality, General Health, Social Functioning, Bodily Pain, Role Emotional, And Mental Health) have not observed any significant difference; and only one study showed that HILT was superior in terms of physical and mental components of SF-36 in the medium term. This may be due to the lack of differences in pain improvement between the two treatment modalities, as the adverse impact of pain on Qol is universal, and this effect covers every stage of life and occurs regardless of the type or source of the pain [58]. According to the World Health Organization (WHO), individuals suffering from persistent pains are four times more likely to suffer from depression or anxiety than those without pain, and twice as much as the difficulty of working [59].

Very low-quality evidence suggests no difference between ESWT and LLLT in improving hand grip strength in the short term for patients with CTS and LE [25, 35, 37]. Devrismel et al. 2014 [35] and Ghasemi et al. 2023 [37] both omitted information on the mean symptom duration of the CTS and LE, limiting our ability to determine the severity of the condition (i.e., acute or chronic). Including patients in their acute phase might introduce bias when investigating the effect of an intervention. Therefore, future studies are needed to report patient stages or the symptom duration to accurately interpret the interventions’ effect.

For ROM, there was no difference between ESWT and laser therapy. The similarity between ROM and function results may be due to the clinical relationship among these variables. It has been reported that there is a high relationship between the ability of the patients to perform function and improvement in ROM for individuals with MSKDs [60]. Although ROM is an important variable, only two of the included studies reported ROM as an outcome. Therefore, it is highly recommended that future studies include ROM as an outcome.

### Limitations

The present review has some limitations that may influence our conclusion. Some studies were not included in the present meta-analysis due to a lack of comparability; thus, not all studies comparing ESWT to laser therapies were represented. Furthermore, despite the growing evidence focusing on the effect of HILT in patients with MSKDs, only a small number of RCTs comparing HILT to ESWT met our inclusion criteria, limiting our ability to draw definitive conclusions about their relative efficacy. Therefore, findings should be approached cautiously.

The overall certainty of the evidence is very low to moderate, due to risk of bias, impression, inconsistency and indirectness. Many trials exhibited concerns or high risks of bias, particularly related to selective outcome reporting and unclear randomization, which may affect the validity of the findings. Also, some studies did not blind participants or therapists, and the allocation sequence was not concealed in most instances, compromising the study’s internal validity. It is worth, however, emphasizing that blinding is not always feasible in physical therapy trials due to the inevitable nature of the treatments.

Moreover, substantial clinical heterogeneity exists among the studies in this review, including variations in pathology, treatment protocols, and symptom durations. As a result, statistical heterogeneity was unavoidable. Notably, even the subgroup analyses did not reduce the heterogeneity, complicating the interpretation of the results and precluding the ability to draw a robust conclusion. Study populations are heterogeneous in terms of MSKDs and symptom duration, responding differently to the treatment protocol; it is; therefore, unclear which individuals would mostly benefit from the treatment. Furthermore, the included interventions were noticeably different between ESWT and laser therapies in terms of intensity, duration, and application site. This diversity further complicates direct comparisons and generalizations of the results. Thus, it remains difficult to recommend effective treatment parameters or generalize the results of ESWT or laser therapies as a whole.

In addition, most trials primarily assessed short-term effects, with limited data on long-term outcomes; thus, it remains unclear if improvements can be sustained over the long run. Finally, less than half of the studies (*n* = 11) explicitly documented adverse events. This raises important questions regarding the long-term safety and efficacy of the treatments. Further high-quality RCTs with larger sample sizes and standardized treatment protocols providing data on the long-term follow-up and potential harms of the interventions are clearly needed to sway the decision between ESWT and laser therapies in treating MSKDs.

## Conclusion

The current meta-analyses suggest no statistically significant difference between ESWT and laser therapy (LLLT and/or HILT) in improving pain, strength, ROM, or Qol in patients with MSKDs. However, ESWT showed a marginal short-term effect in improving functional outcomes compared to LLLT with no significant difference from HILT. Given the very low to moderate quality of evidence, our findings should be approached with caution due to the high risk of bias and heterogeneity among the included studies. The current review highlights the need for further high-quality RCTs, particularly those comparing ESWT to HILT, with larger sample sizes on long-term follow-up to enhance the quality of evidence, validate optimal treatment protocols, and draw more incisive conclusions.

## Electronic supplementary material

Below is the link to the electronic supplementary material.

Supplementary Material 1

Supplementary Material 2

Supplementary Material 3

Supplementary Material 4

Supplementary Material 5

Supplementary Material 6

## Data Availability

No datasets were generated or analysed during the current study.
