# Supplementary material for: Extracorporeal Shock Wave Therapy versus laser therapy in treating musculoskeletal disorders: a systematic review and meta-analysis
Source: Lasers Med Sci. 2025 Apr 15;40(1):194. doi: 10.1007/s10103-025-04392-0 (PMC12000203; doi:10.1007/s10103-025-04392-0)
Supplement: Supplementary file 1 — Supplementary Material 1 [file 10103_2025_4392_MOESM1_ESM.docx]

**Details search strategies:**

|  | Search Date: June 27, 2024  Search update: February 3, 2025 |  |
| --- | --- | --- |
| Database | Search Term | Result |
| PubMed | ("Extracorporeal Shockwave therapy"[All Fields] OR "Extracorporeal shock wave therapy"[All Fields] OR "Extracorporeal shockwave"[All Fields] OR "shock wave therapy"[All Fields] OR "Shockwave therapy"[All Fields] OR "shock wave therapy"[All Fields] OR "ESWT"[All Fields] OR "ECST"[All Fields] OR "ECSW"[All Fields] OR "shock wave"[All Fields] OR ("shockwave"[All Fields] OR "shockwaves"[All Fields])) AND ("high intensity laser therapy"[All Fields] OR "high-intensity laser"[All Fields] OR "High-Power Laser"[All Fields] OR "HILT"[All Fields] OR "low level laser therapy"[All Fields] OR "low-level laser"[All Fields] OR "low-power Laser Therapy"[All Fields] OR "LLLT"[All Fields] OR "low-level laser"[All Fields] OR "Laser Therapy"[All Fields] OR ("low level light therapy"[MeSH Terms] OR ("low level"[All Fields] AND "light"[All Fields] AND "therapy"[All Fields]) OR "low level light therapy"[All Fields] OR "photobiomodulation"[All Fields]) OR "laser"[All Fields]) | 1547 |
| Scopus | TITLE ( ( "Extracorporeal Shockwave therapy" OR "Extracorporeal shock wave therapy" OR "Extracorporeal shockwave" OR "Shock-wave therapy" OR "Shockwave therapy" OR "Shock Wave Therapy" OR "ESWT" OR "ECST" OR "ECSW" OR "shock wave" OR shockwave ) AND ( "high intensity laser therapy" OR "high-intensity laser" OR "High-Power Laser" OR "HILT" OR "low level laser therapy" OR "low level laser" OR "low-power Laser Therapy" OR "LLLT" OR "low-level laser" OR "Laser Therapy" OR photobiomodulation OR laser ) ) | 1497 |
| WOS | **("Extracorporeal Shockwave therapy" OR "Extracorporeal shock wave therapy" OR "Extracorporeal shockwave" OR "Shock-wave therapy" OR "Shockwave therapy" OR "Shock Wave Therapy" OR "ESWT" OR "east" OR "ecsa" OR "shock wave" OR Shockwave) AND ("high intensity laser therapy" OR "high-intensity laser" OR "High-Power Laser" OR "hill" OR "low level laser therapy" OR "low level laser" OR "low-power Laser Therapy" OR "LLLT" OR "low-level laser" OR "Laser Therapy" OR photobiomodulation OR laser)** (Title) | 1066 |
| Cochrane | ("Extracorporeal Shockwave therapy" OR "Extracorporeal shock wave therapy" OR "Extracorporeal shockwave" OR "Shock-wave therapy" OR "Shockwave therapy" OR "Shock Wave Therapy" OR "ESWT" OR "ECST" OR "ECSW" OR "shock wave" OR Shockwave) AND ("high intensity laser therapy" OR "high-intensity laser" OR "High-Power Laser" OR "HILT" OR "low level laser therapy" OR "low level laser" OR "low-power Laser Therapy" OR "LLLT" OR "low-level laser" OR "Laser Therapy" OR photobiomodulation OR laser) in All text | 261 |
| PEDro | Shock wave laser | 329 |
| Google Scholar | allintitle: ("high intensity laser therapy" OR "high-intensity laser" OR "High-Power Laser" OR "HILT" OR "low level laser therapy" OR "low-power Laser Therapy" OR "LLLT" OR "low-level laser" OR "Laser Therapy" OR Photobiomodulation OR laser) AND ("Extracorporeal shockwave therapy" OR "ESWT" OR "Extracorporeal shock wave therapy" OR "Radial Extracorporeal shockwave therapy" OR "ECST" OR "ECSW" OR "shockwave therapy" OR "shock wave therapy") "high intensity laser therapy" OR "high-intensity laser" OR "High-Power Laser" OR "HILT" OR "low level laser therapy" OR "low-power Laser Therapy" OR "LLLT" OR "low-level laser" OR "Laser Therapy" OR Photobiomodulation OR laser AND "Extracorporeal shockwave therapy" OR "ESWT" OR "Extracorporeal shock wave therapy" OR "Radial Extracorporeal shockwave therapy" OR "ECST" OR "ECSW" OR "shockwave therapy" OR "shock wave therapy" | 97 |
