# Supplementary material for: Extracorporeal Shock Wave Therapy versus laser therapy in treating musculoskeletal disorders: a systematic review and meta-analysis
Source: Lasers Med Sci. 2025 Apr 15;40(1):194. doi: 10.1007/s10103-025-04392-0 (PMC12000203; doi:10.1007/s10103-025-04392-0)
Supplement: Supplementary file 2 — Supplementary Material 2 [file 10103_2025_4392_MOESM2_ESM.docx]

**Identification of studies via other methods**

**Identification of studies via databases and registers**

Records identified from:

Reference searching (n = 8)

Citation searching (n = 2)

Records removed *before screening*:

Duplicate records removed (n = 1441)

Records identified from*:

PubMed (n = 1547)

Scopus (n = 1497)

WOS (n = 1066)

Cochrane (n = 261)

Pedro (n = 329)

Google Scholar (n= 97)

**Identification**

Records screened

(n = 3356)

Records excluded**

(n = 3296)

Reports not retrieved

(n = 3)

Reports sought for retrieval

(n = 10)

Reports sought for retrieval

(n = 60)

Reports not retrieved

(n = 12)

**Screening**

Reports assessed for eligibility

(n = 7)

Reports excluded:

Wrong design (n =1)

Language other than English (n = 1)

Poster Presentation (n = 1)

wrong intervention (n = 2)

Reports assessed for eligibility

(n = 48)

Reports excluded: (n= 22)

Trial protocol (n = 1)

Language other than English (n = 3)

Conference abstract (n = 2)

Wrong Population (n= 2)

Wrong intervention (n = 9)

Wrong design (n = 5)

Wrong duration (n = 1)

Studies included in review

(n = 28)

Reports of included studies

(n = 38)

**Included**

PRISMA Flow Diagram
