# Supplementary material for: Extracorporeal Shock Wave Therapy versus laser therapy in treating musculoskeletal disorders: a systematic review and meta-analysis
Source: Lasers Med Sci. 2025 Apr 15;40(1):194. doi: 10.1007/s10103-025-04392-0 (PMC12000203; doi:10.1007/s10103-025-04392-0)
Supplement: Supplementary file 3 — Supplementary Material 3 [file 10103_2025_4392_MOESM3_ESM.docx]

**Population characteristics of the included studies**

| Dropouts | Assessment/Follow up | Diagnosis  Symptom Duration | Mean age (SD)  ESWT T1:  Laser T2:  Control | ESWT/Laser/Control  Gender (M) | ESWT/Laser/Control  (N) | Author  (Year) |
| --- | --- | --- | --- | --- | --- | --- |
| 12 | 16 WKs | PF  NR | 45.05±6.85 **T1**:  44.26±9.53 **T2**: | 16/11 | 19/19 | **Bidoki et al., 2024** |
| 9 | 4 WKs  16 WKs | LE  ≥ 6 MTH | 48.0 – 9.9 **T1**:  48.2 – 9.4 **T2**: | 5/6 | 20/23 | **Celik et al., 2019** |
| 3 | 3 WKs  12 WKs | PF  ≥1 MTH | 45.4 ± 9.7 **T1**:  46.5 ± 10.3 **T2**:  44.0 ± 8.6 **C3**: | 3/6/1 | 25/24/16 | **Cinar et al., 2018** |
| 0 | 7 WKs  15 WKs | LE  NR | 37.76±8.52 **T2**:  40.30±10.00 **T3**: | 8/10 | 30/30 | **Devrimsel et al., 2014** |
| 0 | 4 WKs | Knee OA  NR | 53.9 ± 2.90 **T1**:  53.2 ± 2.39 **T2**: | 5/7 | 15/15 | **El Naggar et al., 2022** |
| 8 | 7 WKs | PF  6.4 ± 5.3/ 5.7 ± 3.5 | 46.0 ± 10.2 **T1**:  46.4 ± 10 **T2**: | 1/ 2 | 23/23 | **Elsehrawy et al., 2018** |
| 0 | 2 WKs  3 WKs | CTS  NR | 41.94±9.73 **T1**:  41.78±6.55 **T2**:  37.44±5.96 **C3**: | 3/ 2 /3 | 18/18/18 | **Ghasemi et al., 2023** |
| 0 | 2 WKs | CTS  NR | 41.9 ± 9.7 **T1**:  41.8 ± 6.6 **T2**: | 3/2 | 18/18 | **Ghasemi et al., 2024** |
| 7 | 3 WKs  16 WKs | SIS  ≥ 4 WKs | 49 (36–65) **T1**:  48 (34–65) **T2**: | 16/10 | 30/34 | **Güloğlu et al., 2021 a** |
| 4 | 3 WKs | Calcaneal spur  ≥4 WKs | 50.0 ± 17.0-63.0 **T1**:  50.0 17.0-65.0 **T2**: | 8/ 6 | 31/31 | **Güloğlu et al., 2021 b** |
| 0 | 3 WKs  15 WKs | MPS  >8 WKs | 57.26±14.31 **T1**:  62.62±9.62 **T2**: | 3/ 4 | 30/31 | **Király et al., 2018** |
| 0 | 6 WKs  8 WKs | SIS  At least 4 weeks | 41.60(3.64) **T1**:  40.27(3.65) **T2**: | NR | 15/15/15 | **Mahmoud et al., 2023** |
| 0 | 4 WKs | Knee OA  last 3 months. | 40.12 (9.45) **T1**:  46.62 (8.68) **T2**: | 9/10 | 20/20 | **Mostafa et al., 2022** |
| 6 | 4 WKs | MPS  NR | 37.6 (12.1) **T1**:  33.0 (10.1) **T2**: | 4/5 | 20/20 | **Özyiğit et al., 2024** |
| 0 | 2 MTH | PF  <6m | 39.66±10.05 **T1**:  38.06 ±12.64 **T2**: | 17 in all group | 15/15/15 | **Riaz et al., 2023** |
| 6 | 1 MTH | PF  NR | 51.5±10.8 in all | 3/2 | 17/17 | **Sanmak et al., 2019** |
| 0 | 2 WKs  1 MTH | MPS  >1‑month | 42.3 ± 10.4 **T1**:  45.3 ± 7.7 **T2**: | 2/1 | 26/20 | **Taheri et al., 2016** |
| 12 | 3 WKs  12 WKs | PF  NR | 53.7±10.1 **T1**:  54.1±8.7 **T2**: | 13/17/CG1:11/CG2:15 | 27/28/CG1:28/CG2:25 | **Takla et al., 2019** |
| 0 | 1 WKs  2 WKs  3 WKs  4 WKs | PF  ESWT: 12.00 (11.25) month  HILT: 8.00 (21.00) months | 48.12 (11.96) **T1**:  46.06 (8.55) **T2**: | 8 in all | 16/16 | **Thammajaree et al., 2023** |
| 0 | 3 WKs | PF  Past month | 46.9(10.9) **T1**:  46.8(10.2) **T2**: | 1/8 | 27/20 | **Timurtas et al., 2022** |
| 6 | 3 WKs  7 WKs | PF  11.0 (12.0) months in HILT  8.0 (6.4) months in ESWT | 47.5 (13.2) **T1**:  51.2 (14.9) **T2**: | 4/11 | 21/25 | **Tongthong et al., 2024** |
| 0 | 5 weeks for ESWT  15 days for LLLT | LE  at least six months. | 48±10 **T1**:  48±11 **T2**: | 14/10 | 26/26 | **Turgay et al., 2020** |
| T1:0  T2:3 | 1 MTH | PF  6 months | 54.45 (6.90) **T1**:  53.40(14.71) **T2**: | 4/4 | 20/17/17 | **Ulusoy et al., 2017** |
| 0 | 2 WKs  6 WKs | LE  3 months | **T1**: 35 ± 8.52  **T2**: 35 ± 8.47  **C3**: 40 ± 10.9 | 7/5/8 | 14/14/14 | **Karaca et al., 2022** |
| T1:5  T2:5 | 3 WKs  12 WKs | LE  NR | **T1:** 47.08 ± 6.05  **T2:** 45.40 ± 8.54 | 9/12 | 25/25 | **Sen et al., 2024** |
| 0 | 4 WKs  3 MTH | Knee OA  More than six months | **T1:**60.43 ± 7.49  **T2:**62.33 ± 9.53  **CG1:**60.66 ± 9.20  **CG2:**60.07 ± 8.05 | 1/5/CG1:2/CG2:4 | 30/30/CG1:30/CG2:30 | **Pasin et al., 2025** |
| 0 | 3 WKs  3 MTH | DQT  NR | **T1**:45.4±7.2  **T2**:42.4±7.8 | 9/7 | 29/31 | **Güngör et al.,**  **2024** |
| T1:3  T2:4 | 3 WKs  9 WKs | LE  Minimum of 3 months | **T1:**45.80 ± 11.65  **T2**:44.35 ± 7.71 | 11/9 | 20/20 | **Bilir et al., 2024** |
| CTS: Carpal tunnel syndrome; C: Control; CG: Control group; DQT: de Quervain tenosynovitis; ESWT: Extracorporeal shock wave therapy; HILT: High Intensity laser therapy; LE: Lateral epicondylitis; LLLT: Low level laser therapy; M: male; MTH: Month; MPS: myofascial pain syndrome; NR: Not Reported; N: number of patients; OA: osteoarthritis; PF: Planter Fasciitis; SIS: Shoulder Impingement Syndrome; SD: Standard Deviation; T: Treatment; WK: Week. | | | | | | |
