# Supplementary material for: Extracorporeal Shock Wave Therapy versus laser therapy in treating musculoskeletal disorders: a systematic review and meta-analysis
Source: Lasers Med Sci. 2025 Apr 15;40(1):194. doi: 10.1007/s10103-025-04392-0 (PMC12000203; doi:10.1007/s10103-025-04392-0)
Supplement: Supplementary file 4 — Supplementary Material 4 [file 10103_2025_4392_MOESM4_ESM.docx]

**
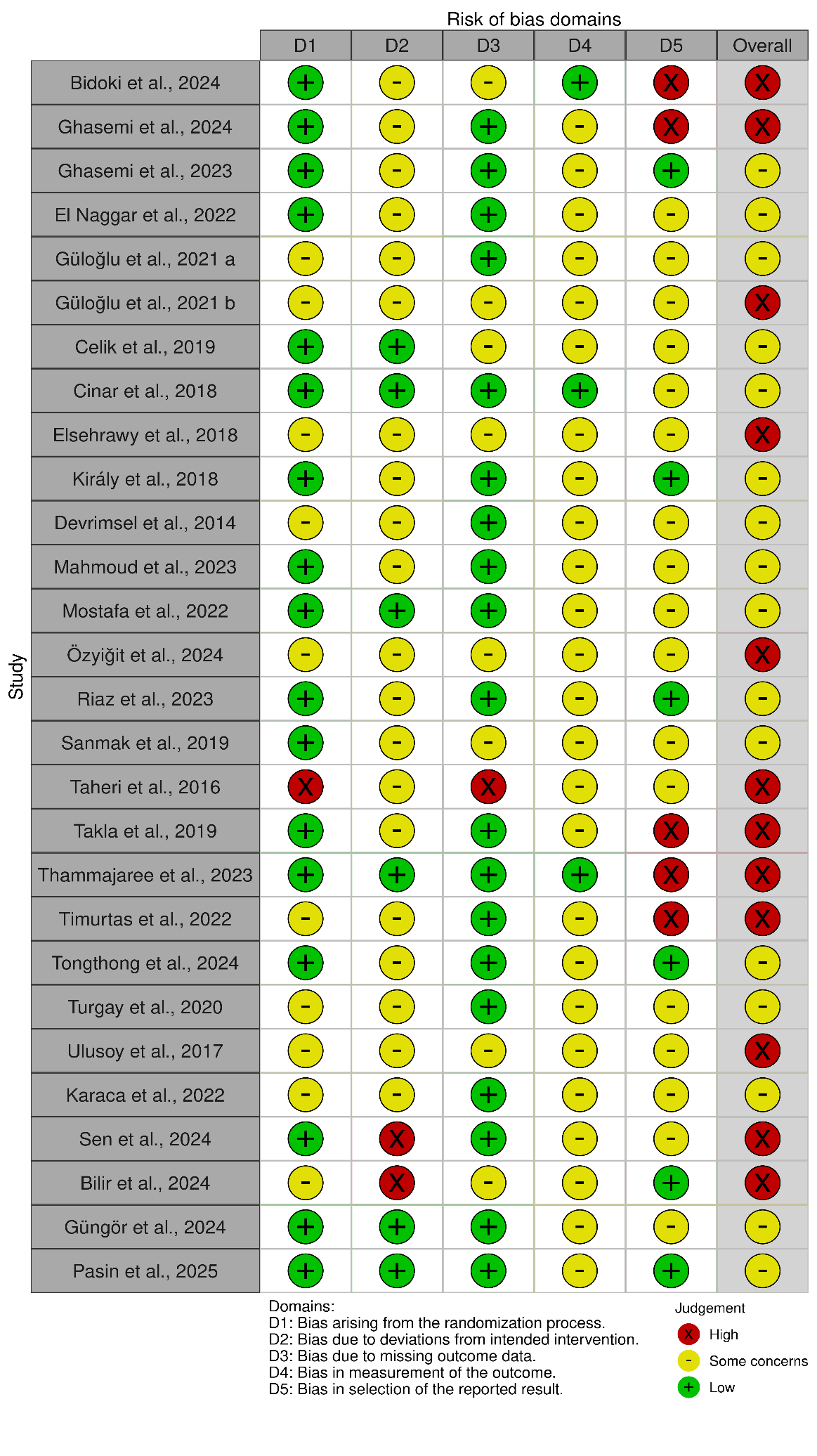
A**

**B
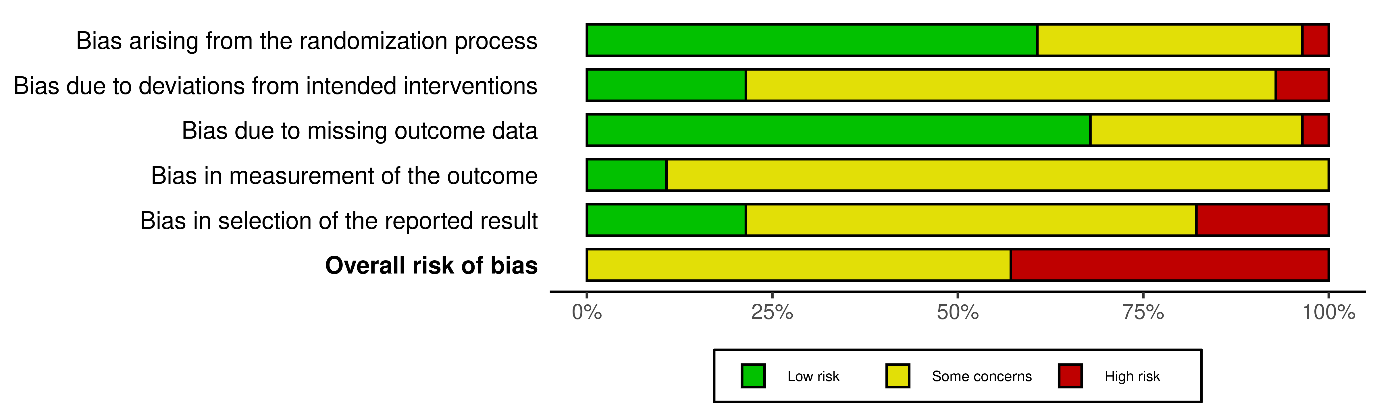
**

**Fig. A** presents details the authors’ assessments of each risk of bias item for each individual study, while **Fig. B** presents the review authors’ judgments about each risk of bias item as percentages across all studies.
