# Supplementary material for: Extracorporeal Shock Wave Therapy versus laser therapy in treating musculoskeletal disorders: a systematic review and meta-analysis
Source: Lasers Med Sci. 2025 Apr 15;40(1):194. doi: 10.1007/s10103-025-04392-0 (PMC12000203; doi:10.1007/s10103-025-04392-0)
Supplement: Supplementary file 5 — Supplementary Material 5 [file 10103_2025_4392_MOESM5_ESM.docx]

**Intervention characteristics of the included studies**

| Adverse events | Outcomes | Laser Parameters   - Type: - Source: - Wavelength (nm): - power (mW): - Energy density (J/cm^2^): - Pulses mode: - Total number of sessions: - Number of sessions per WK: - Site of application: | Shock Wave Parameters   - Type: - Frequency (HZ): - Impulses: - EFD (mJ/mm^2^): - Intensity (bar): - Total number of sessions: - number of sessions per WK: - application: | Control Groups | Study Groups+ Cointervention | Author  (Year) |
| --- | --- | --- | --- | --- | --- | --- |
| SWT: 1 Bruising after treatment | - Quality of life (SF-36 questionnaire) - Heel pain (VAS, HTI) | - HILT - ND: YAG - NR - 30W - 8 J/cm^2^ - NR - 9 sessions - 3 sessions / wk. - Planter fascia area | - Radial ESWT - 12 MHZ (stage 1)/ 15 MHZ (stage 2) - 3000 Pulse (stage1)/ 3000 Pulse (stage 2) - NR - 2-3bar (stage 1)/ 1.8-3 bar (stage 2) - 9 sessions - 3 sessions / wk. - Planter fascia area | None | **T1**: Shock Wave + exercises  **T2**: HILT + exercises | **Bidoki et al., 2024** |
| SWT: pain | - Pain (VAS) - Upper extremity function ((MEPS), (DASH) questionnaire) - Health status and wellbeing (SF-12) - Patient satisfaction (GRC) - Muscle strength (HHD) - Gross grip strength (Jamar HHD**)** | - LLLT - Ga-As - 904nm - 40 mW - 2.4 J/cm^2^ - Continuous - 12 sessions - Three times a week for 4 weeks - lateral epicondyle | - low-dose focused ESWT - NR - 2000 - 0.09mJ/mm^2^ - NR - 4 sessions - Week for 4 weeks - Lateral epicondyle | None | **T1**: Shock Wave + exercises  **T2**: LLLT + exercises | **Celik et al., 2019** |
| NR | - Foot pain (FFI-P, NRS-P) | - LLLT - Ga Al As - 830 nm - 70 mW - 0 to 5.6 j/cm^2^ - Continuous - 10 sessions - 3 times in a week - Plantar fascia at the tenderness points. | - Radial ESWT - NR - 2000 - 0.02mJ/mm^2^ - 1 to 10 bars - 3 sessions - Weekly for 3 weeks - Area of tenderness and planter fascia | CG1: Insole and exercise | **T1**: Shock Wave + Insole and exercise  **T2**: LLLT + Insole and exercise | **Cinar et al., 2018** |
| None | - Elbow pain (SF-MPQ, VAS). - Hand grip strength (Jamar hand dynamometer). | - LLLT - NR - 850 nm - NR - 3.6 J - NR - 10 sessions - NR - Lateral Epicondyle | - RSWT - 16 Hz - 2000 - NR - 1.6 bar - 3 sessions - Three times in 3 weeks - Lateral Epicondyle | None | **T1**: Shock Wave + bandages  **T2**: LLLT + bandages | **Devrimsel et al., 2014** |
| NR | - Pain (VAS) - Knee OA (WOMAC) - Functional mobility (TUG test) | - LLLT - NR - 808 nm - 100 mW - 6 J/cm^2^ - Continuous - 12 sessions - 3 times each week for 4 weeks - Knee joint | - Radial ESWT - 8Hz - 2000 - 0.20 mJ/mm^2^ - NR - 8 sessions - twice per week for four weeks - Knee joint | None | **T1**: Shock Wave + exercises  **T2**: LLLT + exercises | **El Naggar et al., 2022** |
| None | - Heel pain (VAS). - Foot function (FFI) - Plantar fascia thickness (ultrasonography ). | - LLLT - NR - 830 nm - NR - total dose of 27.0 J/cm2 - NR - 6 sessions - 2 procedures/ week during a consecutive 3-week period - Plantar aspect of the heel | - NR - 10.0 Hz - 2050 - NR - 2.5 bars. - Two sessions - Two sessions with one-week interval - Plantar aspect of the heel. | None | **T1**: **S**hock Wave + exercises  **T2**: LLLT + exercises | **Elsehrawy et al., 2018** |
| NR | - Pain (VAS) - Function (BQ) - Hand grip (hand grip dynamometer) - Finger pinch strength (finger dynamometer) | - LLLT - Ga-As - 775 nm - NR - 7 J/cm^2^ - NR - 10 sessions - Five sessions/ week for two consecutive weeks - Carpal tunnel between the distal crease and Kaplan-cardinal line | - Radial ESWT - 15 HZ - 900 - NR - 4 bars - 4 sessions - Two times/ week for two consecutive weeks - Carpal tunnel on the median nerve. | Traditional treatment CG1: | **T1**: Shock Wave + Traditional treatment  **T2**: LLLT + Traditional treatment | **Ghasemi et al., 2023** |
| NR | - Pain (VAS) - Median nerve cross section area (ultrasonography) | - LLLT - Ga-As - 775 nm - NR - 7 J/cm^2^ - NR - 10 sessions - Five sessions/ week for two consecutive weeks - Carpal tunnel between the distal crease and Kaplan-cardinal line | - Radial ESWT - 15 HZ - 900 - NR - 4 bars - 4 sessions - Two times/ week for two consecutive weeks - Carpal tunnel on the median nerve. | None | **T1**: Shock Wave + Traditional treatment  **T2**: LLLT + Traditional treatment | **Ghasemi et al., 2024** |
| None | - Pain (VAS) - ROM (goniometer). - Functional status of the shoulder (SPADI). - Quality of life associated with general health status (Short Form Health Survey). - Severity of depression (BDI). - Patient’s anxiety level (BAI). - Sleep quality and disorder (PSQI). | - LLLT - Ga-Al-As - 1064 nm - NR - 35 J - NR - 15 sessions - Fifteen sessions for 3 weeks, every weekday. - Subacromial space | - NR - NR - 1500 - 0.12 mJ/mm^2^ - 1.5 bar - 3 sessions - Once a week for 3 weeks - Subacromial space | None | **T1**: Shock Wave  **T2**: LLLT | **Güloğlu et al., 2021 a** |
| NR | - Pain (VAS) - Foot function (FFI) | - LLLT - YAG - 904 nm - NR - 8 J/cm^2^ - NR - NR - Five points for a total of 5 min for three weeks - Painful heel area and insertion of the plantar fascia on the medial calcaneal area | - NR - 10 HZ - 2000 - NR - 2.5 bar - 3 sessions - Three sessions at one-week intervals - Painful heel area and insertion of the plantar fascia on the medial calcaneal area | None | **T1**: Shock Wave + Traditional treatment  **T2**: LLLT + Traditional treatment | **Güloğlu et al., 2021 b** |
| None | - Rest/spontaneous pain (VAS) - Functional impairment (NDI) - Quality of life (SF-36) - Need for pain killer medication | - LLLT - NR - NR - 800 mW (Regions around a Trigger point)/ 2000 mW (Trigger point) - 3 J/cm^2^ (Regions around a Trigger point)/ 9 J/cm^2^ (Trigger point) - NR - 15 sessions - Once daily, 15 times (on 15 work days) - Regions of Trigger points and the Trigger points | - NR - 10 HZ (Regions of Trigger point)/ 10 HZ (Trigger point) - 1000 (Regions of Trigger point)/ 1000 (Trigger point) - 0.25 mJ/mm2 (Regions of Trigger point) / 0.25 mJ/mm2 (Trigger point) - 1.5 bar (Regions of Trigger point) / 2 bar (Trigger point) - 3 sessions - Once weekly, three times - Regions of Trigger points and the Trigger points | None | **T1**: Shock Wave  **T2**: LLLT | **Király et al., 2018** |
| NR | - Pain (VAS) - Shoulder pain and disability (SPDI) - ROM (electro goniometer) | - HILT - NR - NR - NR - 4000 J (part 1)/ 4000 J (part 2) / 2000 J (last part) - NR - 12 sessions - 2 sessions/WK - Anterior and posterior joint line of shoulder | - RSWT - 3.2 HZ - 3000 - NR - 4 .0 bar - 6 sessions - One time/ WK - Supraspinatus Tendon | CG1:Traditional treatment | **T1**: Shock Wave + Traditional treatment  **T2**: HILT + Traditional treatment | **Mahmoud et al., 2023** |
| NR | - Pain (VAS) - Physical function (DW6m) - Disability (WOMAC) | - HILT - Nd: YAG - 1064 nm - NR - 1500 mJ/cm^2^ - Pulsed - 12 sessions - 3 sessions/week for 4 week - Anterior, medial and lateral aspects of knee joint and line between tibia and femoral condyles | - Focal Shockwave - NR - 1000 - 0.05 mJ/mm^2^ - NR - 4 sessions - One session/ week for 4 weeks - Tender point on medial tibial plateau of affected knee | None | **T1**: Shock Wave + Traditional treatment  **T2**: HILT + Traditional treatment | **Mostafa et al., 2022** |
| NR | - Pain (VAS) - PPT (algometer) - Quality of life (SF-36) - Depression (BDI) | - LLLT - Ga-As - 904 nm - NR - 1.2 J/cm^2^ - NR - 15 sessions - 5 sessions/week for 3 week - The most sensitive 2 trigger points in the trapezius or paraspinal muscles | - NR - 10-15 HZ - 2000 - NR - 2.0 bar - 3 sessions - Once a week for a total of 3 sessions - The most sensitive 2 trigger points in the trapezius or paraspinal muscles | None | **T1**: Shock Wave + exercises  **T2**: LLLT + exercises | **Özyiğit et al., 2024** |
| None | - Pain (VAS) - Function (FFI) | - HILT - NR - 980 nm - 30 W - 10000 J - NR - 9 sessions - Three times/ week for 3 weeks - Heel, the insertion region of the fascia | - NR - 10 HZ - 2000 - NR - 2.0 bar - 6 sessions - Twice a week, for 3 weeks - Heel, the insertion region of the fascia | CG1:Traditional treatment | **T1**: Shock Wave + Traditional treatment  **T2**: HILT + Traditional treatment | **Riaz et al., 2023** |
| NR | - Pain (VAS) - Function (FFI) - Plantar fascia thickness (ultrasonography) | - LLLT - NR - 685 nm - 30 mW - 2 J/cm^2^ - NR - 12 sessions - Three times/ week for four weeks - Insertion site of the plantar fascia and along the fascia | - NR - 10 HZ - 2000 - NR - 2 bars - 3 sessions - Once/ week for three weeks - Insertion site of the plantar fascia and along the fascia | None | **T1**: Shock Wave  **T2**: LLLT | **Sanmak et al., 2019** |
| NR | - Pain (VAS) - Neck pain (NDI) - Shoulder pain and disability (SPDI) | - LLLT - Ga‑AL‑As - NR - 100 mW - 6 J/cm^2^ - NR - 10 sessions - NR - Upper part of trapezius | - RSWT - 10 HZ - 1000 - 3 J/m^2^ - NR - 3 sessions: - NR - Upper part of trapezius | None | **T1**: Shock Wave + exercises  **T2**: LLLT + exercises | **Taheri et al., 2016** |
| SWT: reddening after application + Pain during application | - Pain intensity (VAS) - Pain sensitivity (PPT) - Functional disability (FFI-d) | - LLLT - Ga Al As - 850 nm (5 laser diodes) - 0.046 W/cm2 - 2.8 J/cm² - Continuous - 9 sessions - 3 times per week for 3 weeks - Heel, and the most tender spot | - Focus Shockwave - 4 HZ - 2000 - 0.02 mJ/mm2 - NR - 3 sessions - Once a week for three consecutive weeks - Heel, and the most tender spot | CG1: ESWT + PBMT  CG2: Sham PBMT | **T1**: Shock Wave + exercises  **T2**: LLLT + exercises | **Takla et al., 2019** |
| None | - Pain (VAS) - Pain at 80 N Pressure (Handheld algometer) - Skin Blood Flow and Temperature (LDF) - Thickness assessment (B-mode ultrasound imaging device) - Function (FFI) | - HILT - Nd: YAG - 1064 nm - 6 W - 5 J/cm^2^ (Initial phase: 60 J/ Intermediate phase: 30 J/ Final phase: 60 J) - Pulsed - 6 sessions - Three weeks (two sessions/week) - Tenderness area of the plantar aponeurosis | - RSWT - 10 HZ - 2000 - NR - 2–3 bars - 6 sessions - Two sessions/week - Tenderness area of the plantar aponeurosis | None | **T1**: Shock Wave  **T2**: HILT | **Thammajaree et al., 2023** |
| NR | - Pain (FFI) - Disability (FFI) - Activity limitation subscales (FFI) | - LLLT - Ga-Al-As - 850nm - 100 mW - 5.6 J/cm^2^ - Continuous - 9 sessions - 3 times for three weeks - Area of tenderness and plantar fascia | - RSWT - NR - 2000 - 0.2 mJ/mm^2^ - 1-10 bar - 3 sessions - Once a week for 3 weeks - Area of tenderness and plantar fascia | None | **T1**: Shock Wave + exercises  **T2**: LLLT + exercises | **Timurtas et al., 2022** |
| None | - Pain (VAS) - Foot function (FAAM) | - HILT - Nd: YAG - 1064 nm - 10.5 W - 360 to 1,780 mJ/cm^2^ - Pulsed - 9 sessions - 3 sessions per week for three weeks - Plantar fascia | - RSWT - 10 HZ - 2000 - 0.38 mJ/mm^2^ - 2 bars - 3 sessions - One session per week for three weeks - Plantar fascia | None | **T1**: Shock Wave + exercises  **T2**: HILT + exercises | **Tongthong et al., 2024** |
| NA | - Pain (VAS) - Limitations of the daily activities (DASH) - Pain level and specific and daily activities (PRTEE) - Quality of life (SF-36) | - LLLT - NR - 905 nm - NR - 0.25-1,2 J per point/area - Pulsed - 15 sessions - 15 sessions on consecutive days - Lateral extensor group of the forearm | - NR - NR - 2000 - NR - NR - 5 sessions - One session/ week for 5 weeks - Common extensor origin of the affected elbow. | None | **T1**: Shock Wave + exercises  **T2**: LLLT + exercises | **Turgay et al., 2020** |
| None | - Pain (VAS) - Functional response (RMS) - Heel sensitivity (HTI) - Functional improvement (AOFAS) - Plantar fascial thickness (MRI) | - LLLT - Ga Al As - 830 nm - 50 mW - 8 J/cm2 - NR - 15 sessions - 5 sessions each week during a consecutive 3-week period - Painful heel | - NR - 10 Hz - 2000 - NR - 2.5 bar - 9 sessions - 3 sessions per week for 3 weeks - Painful heel | CG1:US | **T1**: Shock Wave  **T2**: LLLT | **Ulusoy et al., 2017** |
| NR | - Pain (VAS) - Grip strength (dynamometer) - Function (DHI) + (PRTEE) | - HILT - BTL-6000, UK - NR - Analgesic effect: 4 W   Bio stimulation effect: 6 W   - Analgesic effect: 6 J/cm²   Bio stimulation effect: 12 J/cm²   - NR - 4 sessions - 2 sessions per week - Affected elbow | - NR - 10 Hz - 2000 - NR - 2.5 bars - 4 sessions - 2 sessions per week - affected elbow | CG1: Traditional treatment | **T1**: Shock Wave + Traditional treatment  **T2**: HILT + Traditional treatment | **Karaca et al., 2022** |
| NR | - Pain (VAS) - Grip strength (dynamometer) - Disability (Q-DASH) | - HILT - Nd: YAG - 1064 nm - 15 W - 39 J/cm^2^ - Pulsed - 15 consecutive sessions - Course of 3 weeks - Lateral epicondyle | - RSWT - 8 Hz - 1500 - 0.18 mj/mm^2^ - NR - 3 sessions - once a week for 3 weeks - Lateral epicondyle | None | **T1:** Shock Wave + Traditional treatment  **T2:** HILT + Traditional treatment | **Sen et al., 2024** |
| NR | - Pain (VAS) - Function (WOMAC) - Quality of life (SF-36) - dynamic balance and functional mobility (TUG) | - LLLT - (MLS Laser M6 Robotic Multiwave Lock System, Asalaser, Italy) - 904 nm - 100% power - 3 J - NR - 8 sessions - 2 sessions per week for 4 weeks - Five points on the anterior part of the joint space | - RSWT - 12 Hz - 3000 - NR - 2.5 bars - 3 sessions - 1 session per week - knee joint | CG1: PEMF + exercises  CG2: exercises | **T1:** Shock Wave + exercises  **T2:** LLLT + exercises | **Pasin et al., 2025** |
| NR | - Pain (VAS) - Function (QDASH) - grip strength (dynamometer) - level of social, occupational, and mental functionality (Global Assessment Scale) - PPT ( algometer) | - HILT - BTL-6000; BTL Industries, Ashford, UKNR - NR - 8 W (analgesic) and 4 W (biostimulation) - 6 J/cm² (analgesic) and 120 J/cm² (biostimulation) - NR - 10 sessions - 5 sessions - radial styloid process | - NR - 18 Hz to 21 Hz - 1000 - NR - 1.4 bar to 1.8 bar - 5 sessions - 2 sessions intervals for three weeks - radial styloid process | None | **T1:** Shock Wave  **T2:** HILT | **Güngör et al., 2024** |
| NR | - Pain (VAS) - Function (QDASH) - grip strength (dynamometer) - thickness of the CET (sonographic imaging) | - HILT - HIRO TT (ASA, Italy) - NR - Initial sessions: 4 W,   Subsequent sessions: 6 W   - Initial sessions: 6 J/cm²,   Subsequent sessions: 100-150 J/cm²   - NR - 9 sessions - 3 sessions - Most painful area of the elbow | - NR - 10 Hz - 2000 - NR - 2.5 bar - 3 sessions - 1 session - common extensor origin of the affected elbow | None | **T1:** Shock Wave  **T2:** HILT | **Bilir et al., 2024** |
|  | **AOFAS**: American Orthopedic Foot and Ankle Society ankle-hindfoot scale; **BAI**: Beck anxiety inventory; **BDI**: Beck depression inventory; **BTL**: Bio Therapeutic Laser; **BQ**: Boston carpal tunnel syndrome questionnaire; **CET:** common extensor tendon; **CG**: Control group; **DASH**: Disabilities of the arm, shoulder, and hand; **DW6m**: 6-min walking test; **EFD**: Energy Flux Density; **ESWT**: Extracorporeal shock wave therapy; **FAAM**: Foot and Ankle Ability Measure; **FFI**: Foot function index; **FFI-d**: functional foot index disability subscale; **FFI-P**: Foot function index pain subscale; **Ga-AL-As**: Gallium aluminum arsenide; **Ga-As**: Gallium arsenide; **GRC**: Global rating of change; **HHD**: Hand-held dynamometer; **HTI**: heel tenderness index; **HILT**: High Intensity laser therapy; **LDF**: Laser Doppler flowmetry; **LLLT**: Low level laser therapy; **MRI**: Magnetic Resonance Imaging; **MEPS**: Mayo Elbow Performance Score; **MTH**: Month; **NDI**: Neck disability index; **Nd:YAG**: Neodymium-doped Yttrium Aluminum Garnet; **NR**: Not Reported; **NRS-P**: Numeric rating scale for pain; **OA**: osteoarthritis; **PRTEE**: Patient-Related Lateral Epicondyle-litis Evaluation; **PSQI**: Pittsburgh sleep quality index; **PPT**: Pressure pain threshold;  **Q-DASH**: quick version of the Disabilities of the Arm, Shoulder and Hand scale; **RSWT**: Radial Shock Wave Therapy; **RMS**: Roles–Maudsley score; **SF-MPQ**: Short-form McGill pain questionnaire; **SF-12**: 12-Item Short Form; **SF-36 questionnaire**: 36-question quality of life questionnaire; **SPADI:** Shoulder Pain and Disability Index; **SWT**: Shock wave therapy; **SPDI**: Shoulder Pain and Disability Index; **TUG**: Timed up and go; **T**: Treatment; **US**: ultrasound; **VAS**: Visual analogue scale; **WK**: Week; **WOMAC**: Western Ontario McMaster Universities osteoarthritis Index; **YAG**: Yttrium aluminum garnet. | | | | | |
