# Supplementary material for: Extracorporeal Shock Wave Therapy versus laser therapy in treating musculoskeletal disorders: a systematic review and meta-analysis
Source: Lasers Med Sci. 2025 Apr 15;40(1):194. doi: 10.1007/s10103-025-04392-0 (PMC12000203; doi:10.1007/s10103-025-04392-0)
Supplement: Supplementary file 6 — Supplementary Material 6 [file 10103_2025_4392_MOESM6_ESM.docx]

**GRADE evidence profile of ESWT vs LLLT**

| **N/ study design** | **Condition** | **Risk of bias** | **Inconsistency** | **Indirectness** | **Imprecision** | **Outcome** | **Timepoint** | **N (LLLT group)** | **N (ESWT group)** | **Effect (absolute risk**  **95%CI)** | **Quality of evidence**  **(GRADE)** | **Importance** |
| --- | --- | --- | --- | --- | --- | --- | --- | --- | --- | --- | --- | --- |
| 2 (RCT) | LE | serious^a^ | serious^b^ | not serious | serious^c^ | Pain | Short-term | 56 | 56 | SMD **0.51 SD lower** (1.29 lower to 0.27 higher) | ⨁◯◯◯ Very low | CRITICAL |
| 2 (RCT) | MPS | serious^d^ | not serious | not serious | serious^c^ | Pain | Short-term | 40 | 46 | SMD **0.22 SD higher** (0.2 lower to 0.65 higher) | ⨁⨁◯◯ Low | CRITICAL |
| 5 (RCT) | PF | serious^a^ | serious^b^ | not serious | serious^c^ | Pain | Short-term | 109 | 112 | SMD **0.21 SD lower** (1.33 lower to 0.91 higher) | ⨁◯◯◯ Very low | CRITICAL |
| 2 (RCT) | CTS | serious^e^ | not serious | not serious | serious^c^ | Pain | Short-term | 36 | 36 | SMD **0.44 SD lower** (0.91 lower to 0.03 higher) | ⨁⨁◯◯ Low | CRITICAL |
| 2 (RCT) | KOA | serious^a^ | not serious | not serious | serious^c^ | Pain | Short-term | 45 | 45 | SMD **0.16 SD lower** (0.57 lower to 0.26 higher) | ⨁⨁◯◯ Low | CRITICAL |
| 2 (RCT) | PF | not serious | serious^b^ | not serious | serious^c^ | Pain | Medium-term | 52 | 50 | SMD **0.77 SD lower** (4.02 lower to 2.48 higher) | ⨁⨁◯◯ Low | CRITICAL |
| 10 (RCT) | (KOA (N = 2), LE (N = 2), MPS (N = 2), PF (N = 2, CS (N = 1), SIS (N = 1)) | serious^a^ | serious^b^ | serious^f^ | serious^c^ | Function | Short-term | 253 | 257 | SMD **0.28 SD lower** (0.55 lower to 0.005 lower) | ⨁◯◯◯ Very low | CRITICAL |
| 3 (RCT) | (MPS (N = 1), LE (N = 1), (KOA = 1)) | serious^e^ | not serious | serious^g^ | serious^c^ | Quality of life (Role Physical, Role Emotional) | Short-term | 87 | 86 | -Role Physical: MD **12.01 higher** (1.87 higher to 22.15 higher)  - Role Emotional: MD **8.43 higher** (0.77 lower to 17.63 higher) | ⨁◯◯◯ Very low | CRITICAL |
| 3 (RCT) | (MPS (N = 1), and LE (N = 1), (KOA = 1)) | serious^e^ | serious^b^ | serious^g^ | serious^c^ | Quality of life (Physical Functioning, Mental Health, Energy/Fatigue, General Health, Social Functioning, and Pain) | Short-term | 87 | 86 | -Physical Functioning: MD **3.84 higher** (6.12 lower to 13.81 higher)  -Bodily Pain: MD **7.10 higher** (5.58 lower to 19.77 higher)  -General Health: MD **4.3 higher** (3.44 lower to 12.04 higher)  -Vitality: MD **2.21 higher** (7.60 lower to 12.02 higher)  -Social Functioning: MD **8.42 higher** (1.04 lower to 17.87 higher)  -Mental Health: MD **2.85 higher** (5.19 lower to 10.89 higher) | ⨁◯◯◯ Very low | CRITICAL |
| 3 (RCT) | (LE (N= 2), CTS (N= 1)) | not serious | serious^b^ | serious^h^ | serious^c^ | Grip Strength | Short-term | 74 | 74 | MD **2.38 higher** (0.96 lower to 5.73 higher) | ⨁◯◯◯ Very low | IMPORTANT |
| 1 (RCT) | SIS | not serious | not serious | not serious | serious^c^ | ROM | Short-term | 34 | 30 | Not Pooled | ⨁⨁⨁◯ Moderate | IMPORTANT |

**CI:** Confidence interval; **CS:** calcaneal spur; **CTS:** Carpal tunnel syndrome; **ESWT:** Extracorporeal shock wave therapy; **KOA:** Knee osteoarthritis; **LE:** Lateral epicondylitis; **LLLT:** Low level laser therapy; **MD:** Mean difference; **MPS:** Myofascial pain syndrome; **N:** Number of studies; **PF:** planter fasciitis; **RCT:** Randomized clinical trials; **ROM:** Range of motion; **SD:** Standard deviation; **SIS:** Shoulder Impingement Syndrome; **SMD:** Standardized mean difference.

**Explanations**

a. Most information is from studies at some concerns risk of bias.

b. Inconsistency: Serious, I2 > 50%

c. sample size less than 800

d. The proportion of information from studies at high risk of bias is sufficient to affect the interpretation of results.

e. Potential limitations are likely to lower confidence in the estimate of effect.

f. The present systematic review aimed to assess the efficacy of ESWT vs laser therapy on MSK disorders. There are differences in study population (KOA (N = 2), LE (N = 2), MPS (N = 2), PF (N = 2, CS (N = 1), SIS (N = 1)).

g. The present systematic review aimed to assess the efficacy of ESWT vs laser therapy on MSK disorders. There are differences in study population (MPS (N = 1), and LE (N = 1), (KOA = 1)).

h. The present systematic review aimed to assess the efficacy of ESWT vs laser therapy on MSK disorders. There are differences in study population (LE (N= 2), CTS (N= 1)).

| **N/ study design** | **Condition** | **Risk of bias** | **Inconsistency** | **Indirectness** | **Imprecision** | **Outcome** | **Timepoint** | **N (HILT group)** | **N (ESWT group)** | **Effect (absolute risk**  **95%CI)** | **Quality of evidence**  **(GRADE)** | **Importance** |
| --- | --- | --- | --- | --- | --- | --- | --- | --- | --- | --- | --- | --- |
| 3 (RCT) | PF | not serious | serious^b^ | not serious | serious^c^ | Pain | Short-term | 56 | 52 | MD **0.38 lower** (1.71 lower to 0.94 higher) | ⨁⨁◯◯ Low | CRITICAL |
| 3 (RCT) | LE | serious^a^ | not serious | not serious | serious^c^ | Pain | Short-term | 59 | 59 | MD **0.65 higher** (0.26 higher to 1.04 higher) | ⨁⨁◯◯ Low | CRITICAL |
| 8 (RCT) | (KOA (N= 1), SIS (N= 1), PF (N= 2), LE (N= 3), DQT (N = 1)) | serious^a^ | serious^b^ | Serious^d^ | serious^c^ | Function | Short-term | 156 | 154 | SMD **0.44 SD higher** (0.07 lower to 0.96 higher) | ⨁◯◯◯ Very low | CRITICAL |
| 1(RCT) | PF | not serious | not serious | not serious | serious^c^ | Quality of life | Medium-term | 19 | 19 | Not Pooled | ⨁⨁⨁◯ Moderate | CRITICAL |
| 2 (RCT) | LE (N= 2) | not serious | not serious | not serious | serious^c^ | Grip Strength | Short-term | 39 | 39 | MD **1.32 lower** (5.10 lower to 2.46 higher) | ⨁⨁⨁◯ Moderate | IMPORTANT |
| 1 (RCT) | SIS | not serious | not serious | not serious | serious^c^ | ROM | Short-term | 15 | 15 | Not Pooled | ⨁⨁⨁◯ Moderate | IMPORTANT |

**GRADE evidence profile of ESWT vs HILT**

**CI:** Confidence interval; **CTS:** Carpal tunnel syndrome; DQT: de Quervain tenosynovitis; **ESWT:** Extracorporeal shock wave therapy; **HILT:** High-Intensity Laser Therapy; **KOA:** Knee osteoarthritis; **LE:** Lateral epicondylitis; **MD:** Mean difference; **N:** Number of studies **PF:** planter fasciitis; **RCT:** Randomized clinical trials; **SD:** Standard deviation; **SIS:** Shoulder Impingement Syndrome; **SMD:** Standardized mean difference; **ROM:** Range of motion

#### **Explanations**

a. The proportion of information from studies at high risk of bias is sufficient to affect the interpretation of results.

b. Inconsistency: Serious, I2 > 50%

c. sample size less than 800

d. The present systematic review aimed to assess the efficacy of ESWT vs laser therapy on MSK disorders. there are differences in study population (KOA (N= 1), SIS (N= 1), PF (N= 2), LE (N= 3), Qe Quervain tenosynovitis (N = 1))
